# Author Correction: Complex de novo structural variants are an underestimated cause of rare disorders

**DOI:** 10.1038/s41467-026-69646-z

**Published:** 2026-02-17

**Authors:** Hyunchul Jung, Tsun-Po Yang, Susan Walker, Petr Danecek, O. Isaac Garcia-Salinas, Matthew D. C. Neville, Joseph Christopher, Isidro Cortés-Ciriano, Helen Firth, Aylwyn Scally, Matthew Hurles, Peter Campbell, Raheleh Rahbari

**Affiliations:** 1 Wellcome Sanger Institute, Wellcome Genome Campus, Hinxton, UK; 2 Genomics England, London, UK; 3 Department of Clinical Genetics, Cambridge University Hospitals, Cambridge, UK; 4 Department of Genomic Medicine, University of Cambridge, Cambridge, UK; 5 European Molecular Biology Laboratory, EBI, Hinxton, Cambridge, UK; 6 Department of Genetics, University of Cambridge, Downing Street, Cambridge, UK

**Keywords:** Disease genetics, Genome informatics, Structural variation

Correction to: *Nature Communications* 10.1038/s41467-025-64722-2, published online 3 November 2025

In the version of this article initially published, there was a typographical error in the second paragraph of the Discussion section, where in the text now reading “In addition, 66 of 145 (46%) pathogenic SVs identified in our study were balanced rearrangements,” 66 of 145 (46%) originally read “51 of 151 (34%).” In Fig. 2g, the gene label “*SRRM2*” was mistakenly placed to the right of the *x*-axis and is now realigned toward the center. In the first paragraph of the Results section, a related reference and associated discussion were missing. The text is now amended to include “Furthermore, 19 of the pathogenic dnSVs involving inversions were validated by an independent group [Pagnamenta, A. T. et al. The impact of inversions across 33,924 families with rare disease from a national genome sequencing project. *Am. J. Hum. Genet*. 10.1016/j.ajhg.2024.04.018 (2024)].” The changes are made in the HTML and PDF versions of the article.

