# Supplementary material for: Complex de novo structural variants are an underestimated cause of rare disorders
Source: Nat Commun. 2025 Nov 3;16:9528. doi: 10.1038/s41467-025-64722-2 (PMC12583596; doi:10.1038/s41467-025-64722-2)
Supplement: Supplementary file 1 — Supplementary Information [file 41467_2025_64722_MOESM1_ESM.pdf]

Supplementary Figures

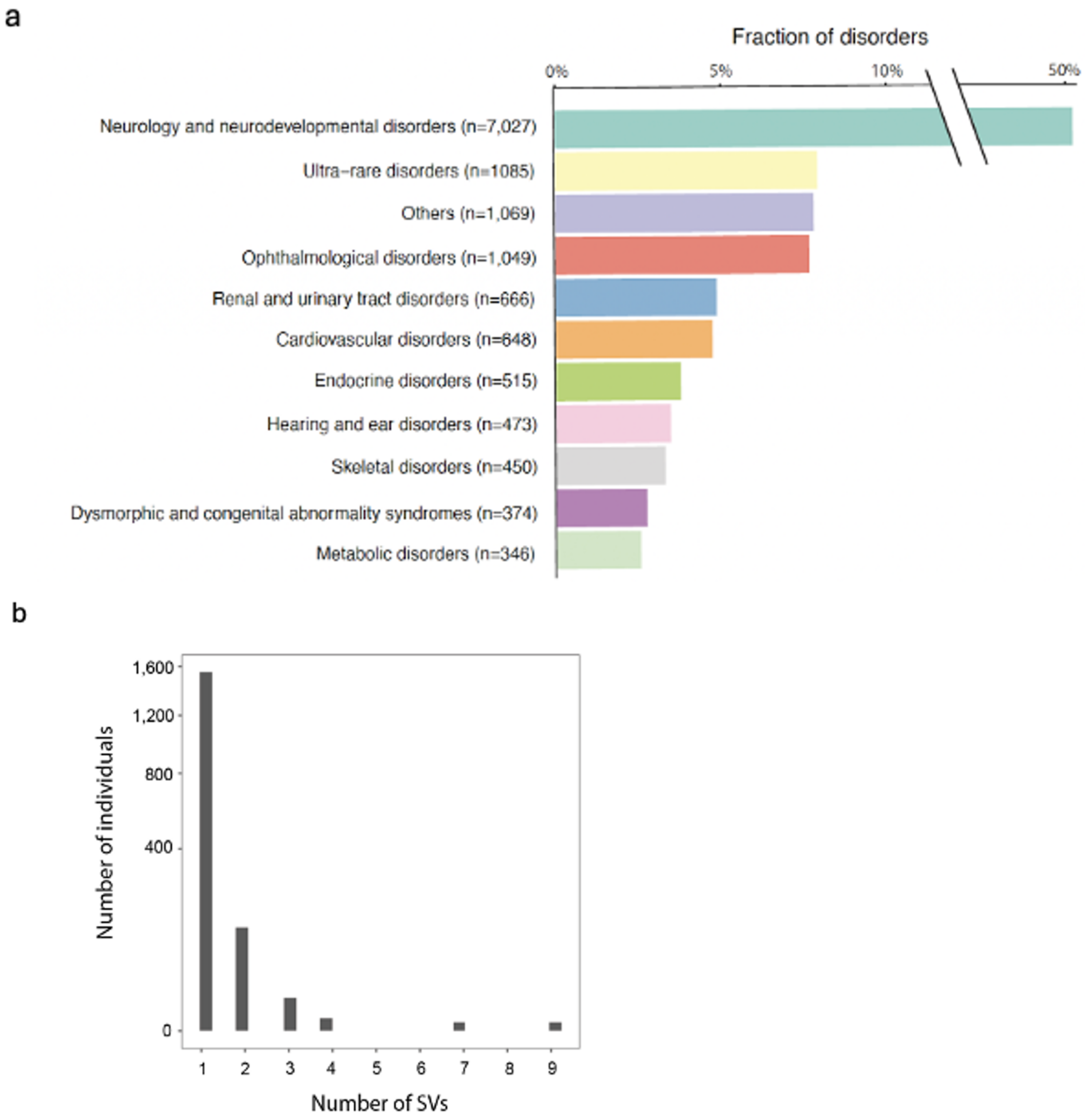

Figure S1. Summary statistics of GEL data. (**a**) Proportion of samples by rare disorder types. (**b**) Histogram of the number of individuals (y axis) by the number of de novo SVs (x axis) in the cohort.

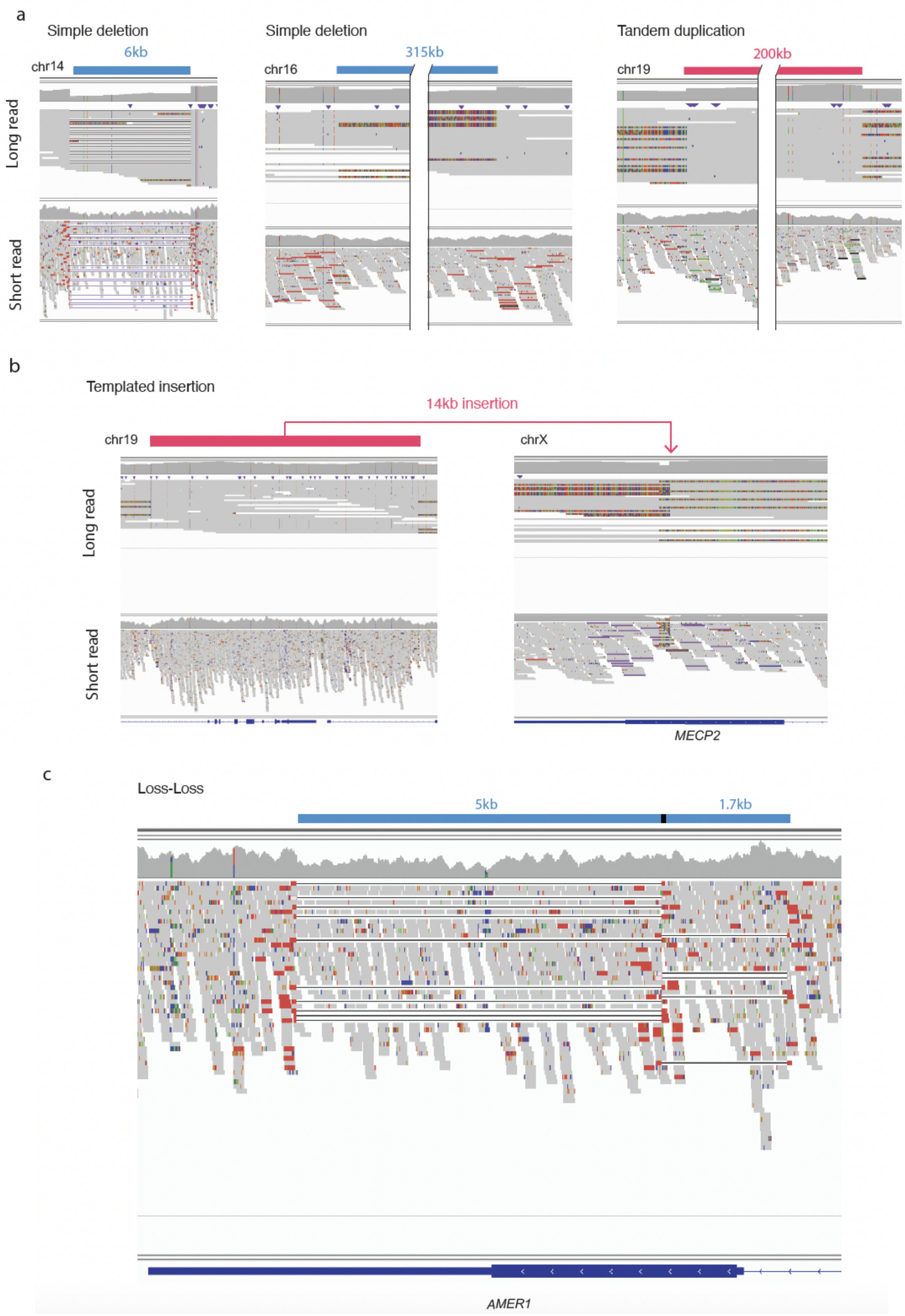

Figure S2. Validation of simple (a-b) and complex dnSVs (c) by long-read sequencing.

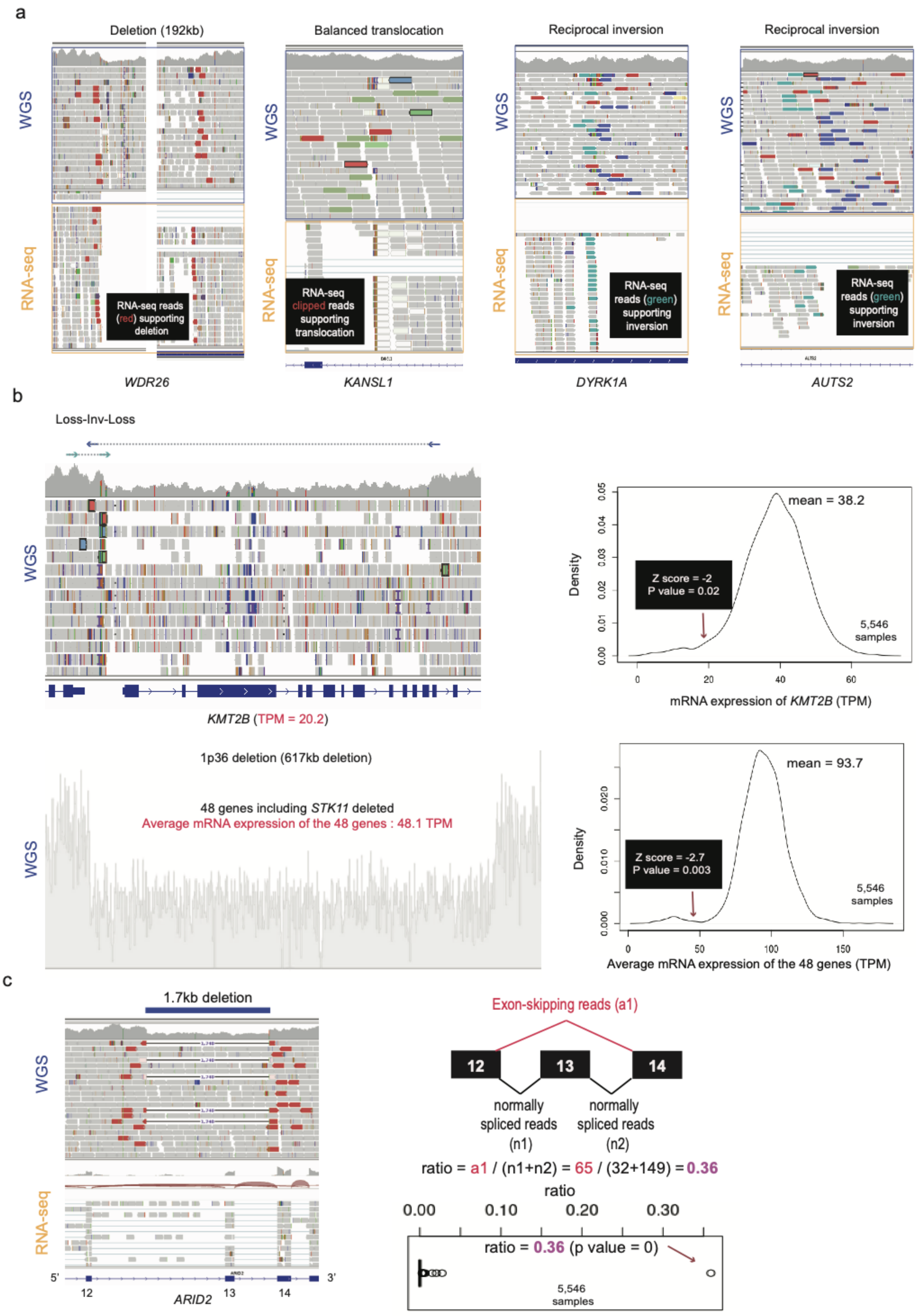

a
Deletion (192kb)
Balanced translocation
Reciprocal inversion
Reciprocal inversion
WGS
RNA-seq
RNA-seq reads (red) supporting deletion
RNA-seq clipped reads supporting translocation
RNA-seq reads (green) supporting inversion
RNA-seq reads (green) supporting inversion
WDR26
KANSL1
DYRK1A
AUTS2
b
Loss-Inv-Loss
KMT2B (TPM = 20.2)
mean = 38.2
Z score = -2
P value = 0.02
5,546 samples
Density
mRNA expression of KMT2B (TPM)
1p36 deletion (617kb deletion)
48 genes including STK11 deleted
Average mRNA expression of the 48 genes : 48.1 TPM
mean = 93.7
Z score = -2.7
P value = 0.003
Average mRNA expression of the 48 genes (TPM)
c
1.7kb deletion
ARID2
5'
3'
12
13
14
Exon-skipping reads (a1)
normally spliced reads (n1)
normally spliced reads (n2)
ratio = a1 / (n1+n2) = 65 / (32+149) = 0.36
ratio
0.00
0.10
0.20
0.30
ratio = 0.36 (p value = 0)
5,546 samples

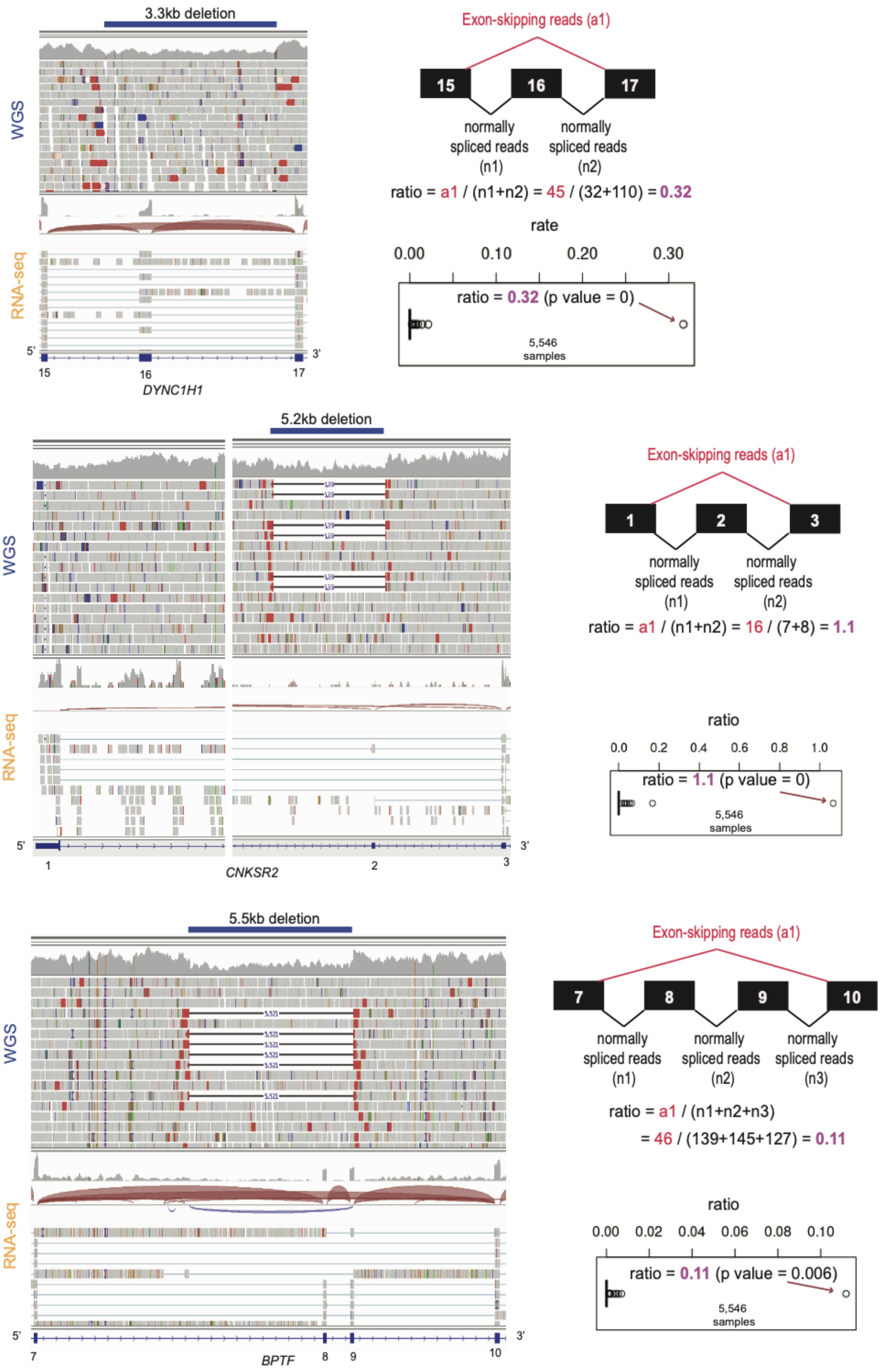
3.3kb deletion
WGS
RNA-seq
5'
3'
15
16
17
DYNC1H1
Exon-skipping reads (a1)
15
16
17
normally spliced reads (n1)
normally spliced reads (n2)
ratio = a1 / (n1+n2) = 45 / (32+110) = 0.32
rate
0.00
0.10
0.20
0.30
ratio = 0.32 (p value = 0)
5,546 samples
5.2kb deletion
WGS
RNA-seq
5'
3'
1
CNKSR2
2
3
Exon-skipping reads (a1)
1
2
3
normally spliced reads (n1)
normally spliced reads (n2)
ratio = a1 / (n1+n2) = 16 / (7+8) = 1.1
ratio
0.0
0.2
0.4
0.6
0.8
1.0
ratio = 1.1 (p value = 0)
5,546 samples
5.5kb deletion
WGS
RNA-seq
5'
3'
7
BPTF
8
9
10
Exon-skipping reads (a1)
7
8
9
10
normally spliced reads (n1)
normally spliced reads (n2)
normally spliced reads (n3)
ratio = a1 / (n1+n2+n3)
= 46 / (139+145+127) = 0.11
ratio
0.00
0.02
0.04
0.06
0.08
0.10
ratio = 0.11 (p value = 0.006)
5,546 samples

Figure S3. Validation of pathogenic dnSVs using RNA-seq. (a) Evidence of WGS (top panel) and RNA-seq reads (bottom panel) supporting dnSVs. (b) Validation of underexpression of genes hit by dnSVs. The average expression of 41 genes within the deletion (i.e., 1p36) was computed (bottom), and then the degree of significance of underexpression was estimated using 5,546 background samples in Genomics England. (c) Validation of abnormal splicing patterns caused by dnSVs. The ratio was computed using abnormally- (nominator) and normally spliced reads (denominator), and then the degree of significance of the ratio was calculated using 5,546 background samples in Genomics England.

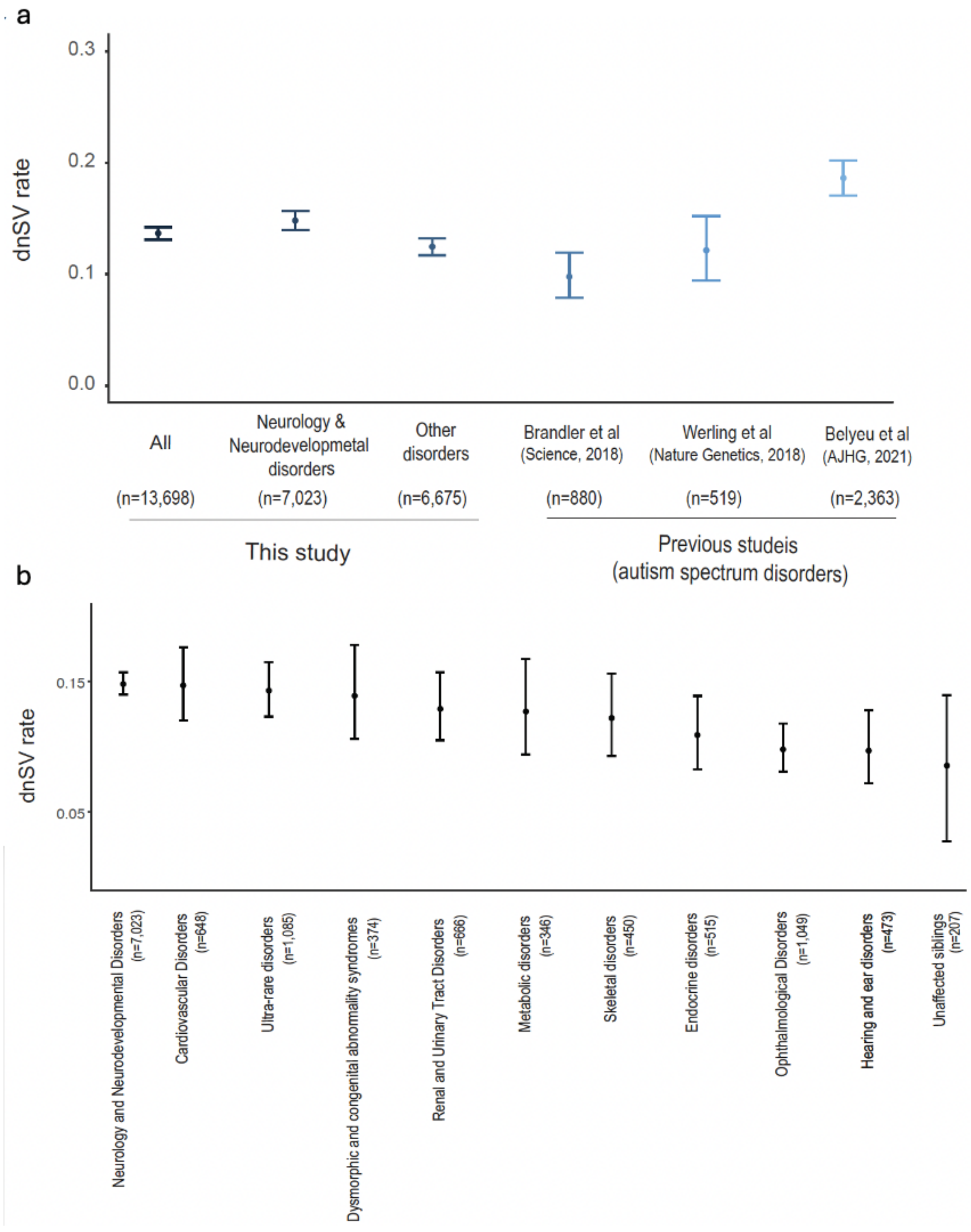

Figure S4. dnSV rate (**a**) Comparison of the rate of dnSVs between our study and previous three studies. The confidence interval was computed using an exact binomial test. (**b**) dnSV rate across rare disorder types. Rare disorder types with data from ≥300 samples available, except for the 'Unaffected siblings' category.

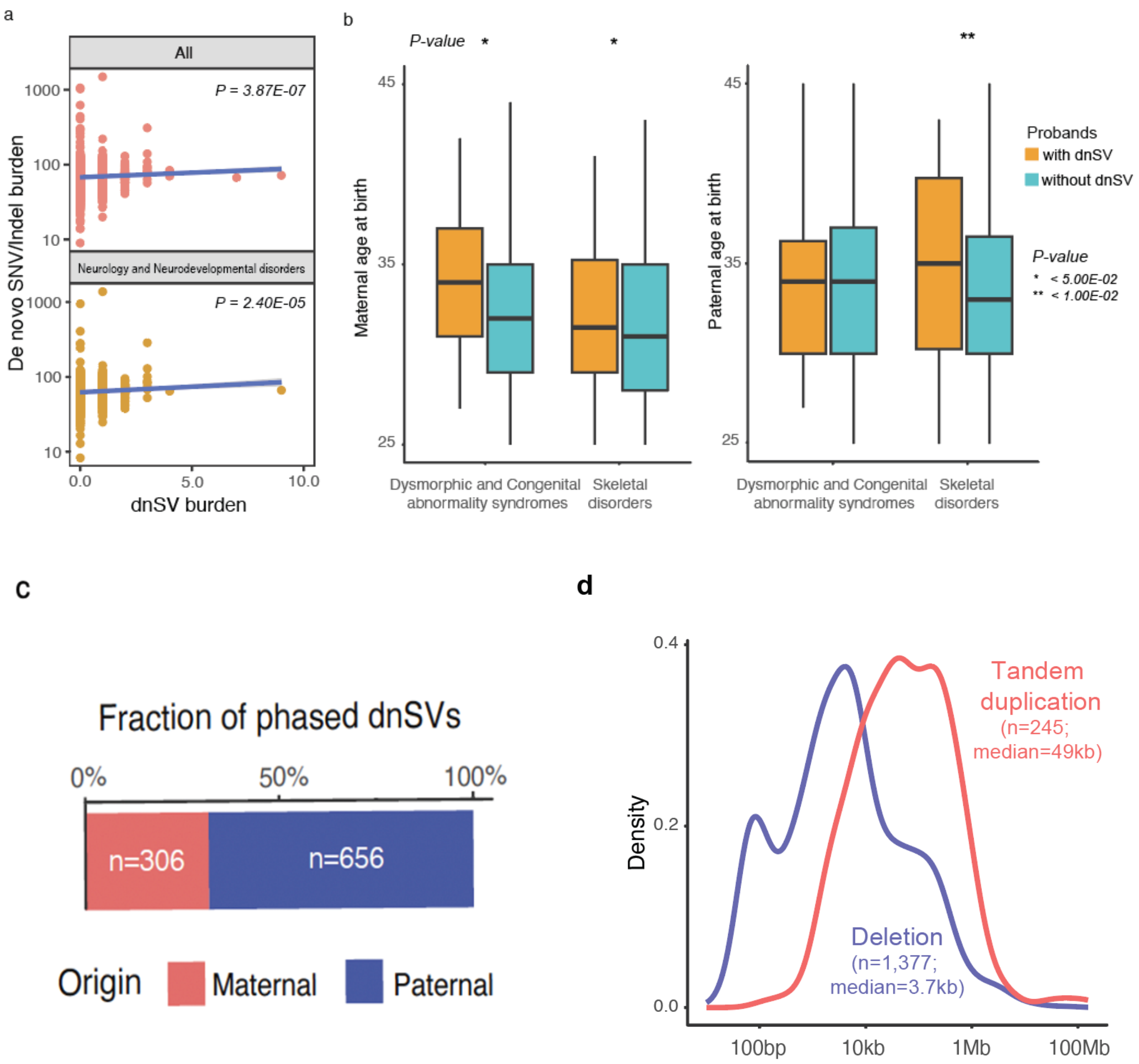

Figure S5. Analysis of dnSV (**a**) Correlation analysis between dnSV and dnSNV/indel burden using all (top panel) and NN samples (bottom panel). The P values were determined based on Spearman correlation analysis (**b**) Comparison of maternal and paternal age at birth between probands with dnSVs and those without dnSVs in Dysmorphic and Congenital abnormality syndromes and skeletal disorders. The P values were calculated based on a one-sided t-test. (**c**) Fraction of phased dnSVs by parental origin. (**d**) Size distribution of simple deletion and tandem duplication.

		Father		
		RR	RV	VV
Mother	RR		RVV : Father<br>RRV : Mother	RVV : Father<br>RRV : Mother
	RV	RRV : Father<br>RRV : Mother_meiosis_I<br>RVV : Mother_meiosis_II		RVV : Father<br>RVV : Mother_meiosis_I<br>RRV : Mother_meiosis_II
	VV	RRV : Father<br>RVV : Mother	RRV : Father<br>RVV : Mother	

Figure S6. Informative genotypes to infer the timing of duplications of maternal origin. A set of informative genotypes to infer the timing of duplications (i.e., meiosis I or II). The genotypes in the green boxes were first used to infer duplications with maternal origin and then those in the orange boxes were utilized to classify the timing of triplication into meiosis I and II. “R” and “V” represent “Reference” and “Variant” alleles, respectively.

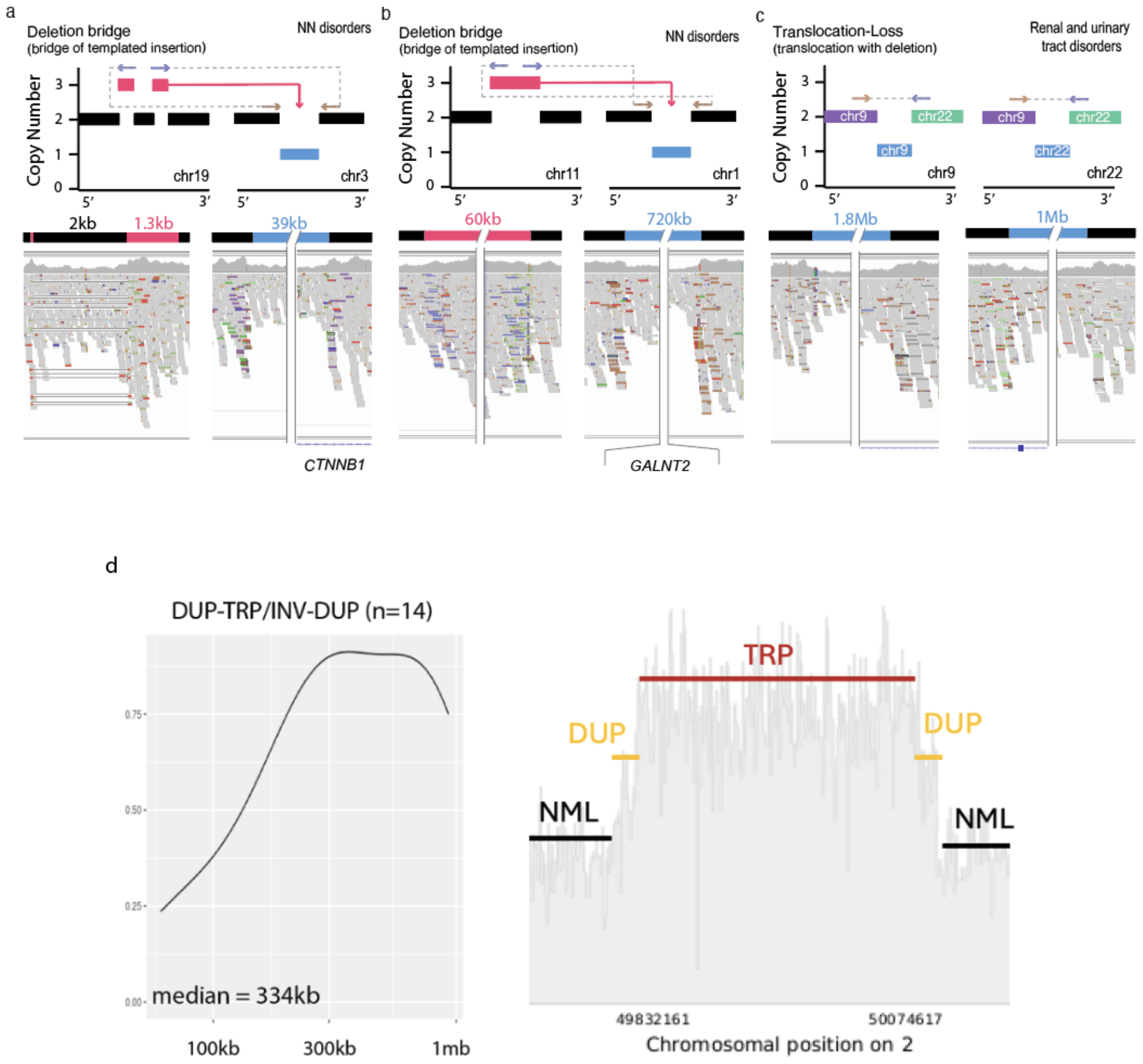

Figure S7. **'Deletion bridge', 'Translocation-Loss' and 'DUP-TRP/INV-DUP'. (a-b)** Representative cases with 'Deletion bridge'. Additional small deletion (size=2kb) in chr19 was found in the templated sequence (**a**). (**c**) Translocation accompanying deletions on both chromosomes is shown. (**d**) Size distribution of DUP-TRP/INV-DUP identified in our cohort (left) and an example of DUP-TRP/INV-DUP with a length of 303kb (right).

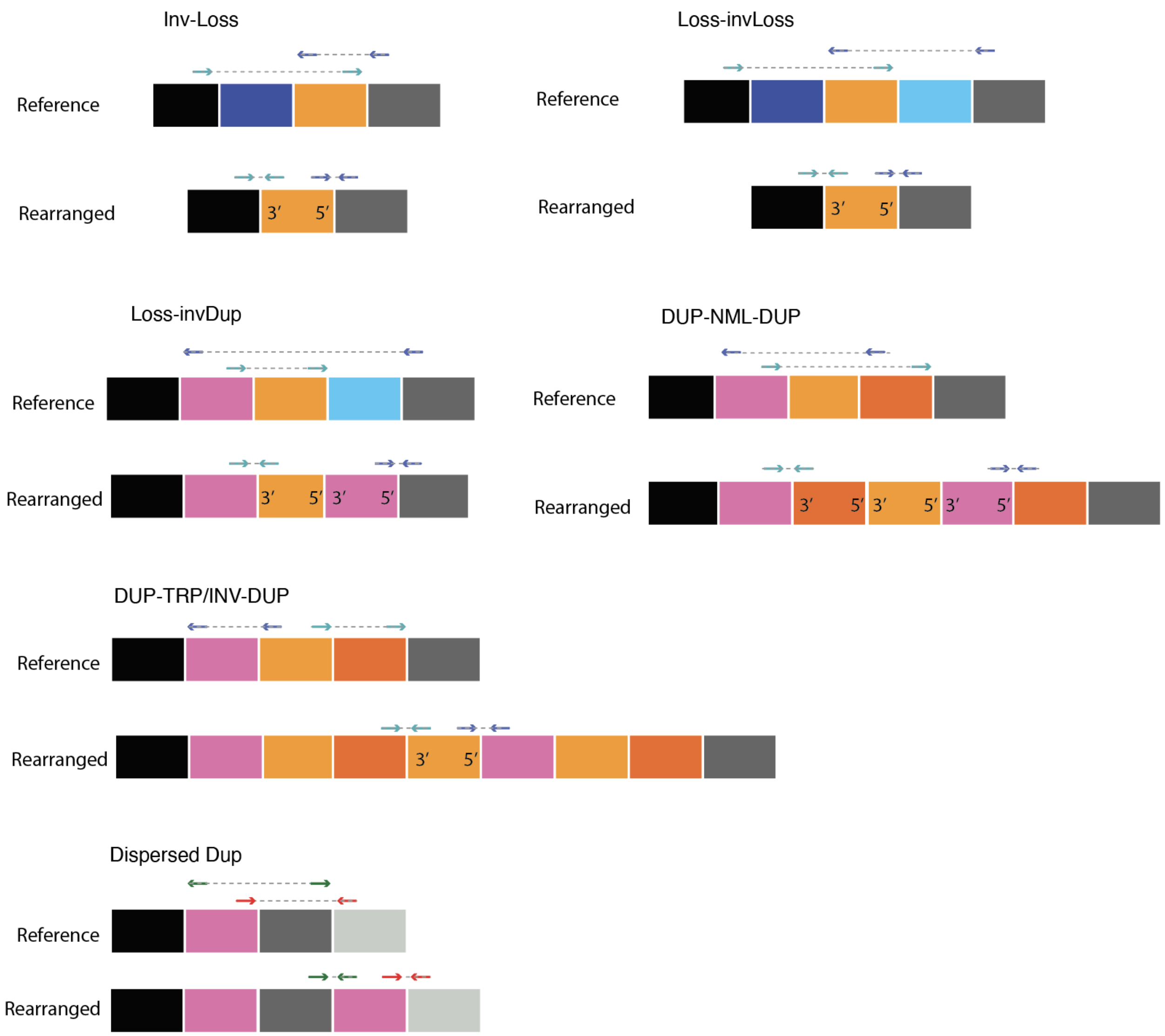

Figure S8. Schematic of representative complex dnSVs classes. The bottom rearranged segment shows one of the possible genomic configurations resulting from each dnSVs class. Note that all possible schematic configurations for each class are not shown.

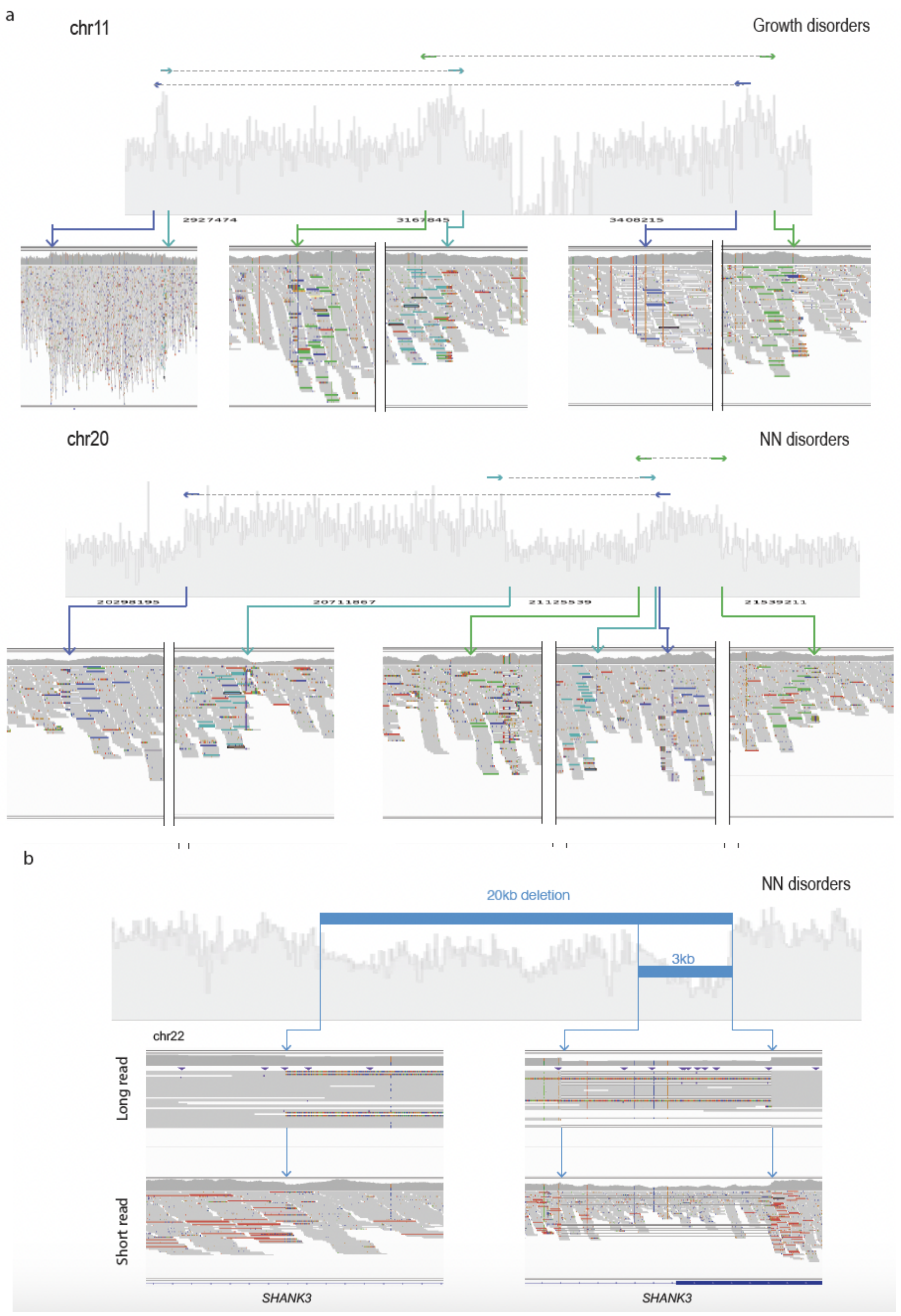

Figure S9. Representative cases with complex SVs. (**a**) DUP-NML-DUP-NML-DUP (**b**) Unclassified complex SV type.

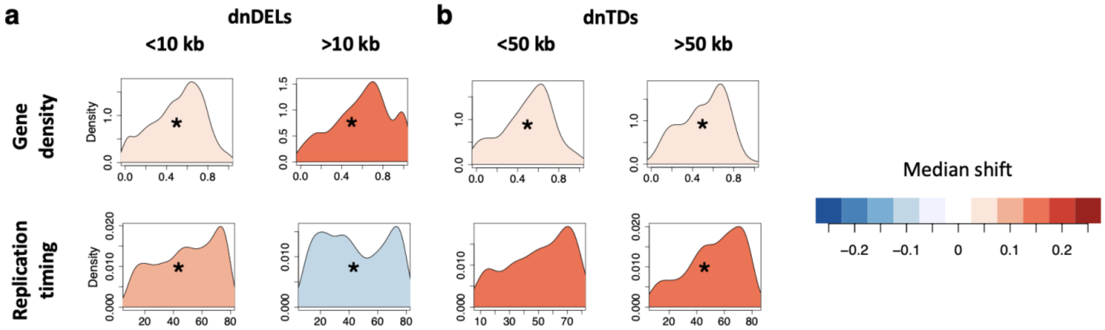

Figure S10. Genomic properties of dnSVs. **(a-b)** Distribution of dnDELs (a) and *de novo* tandem duplications (b) in the context of gene density (top) and replication timing (bottom). P values were calculated using Monte Carlo simulation test (*P < 1E-03). Each density plot is coloured by the extent of the median shift below (blue) or above (red) 0.5, assuming that a uniform distribution has a median of 0.5.

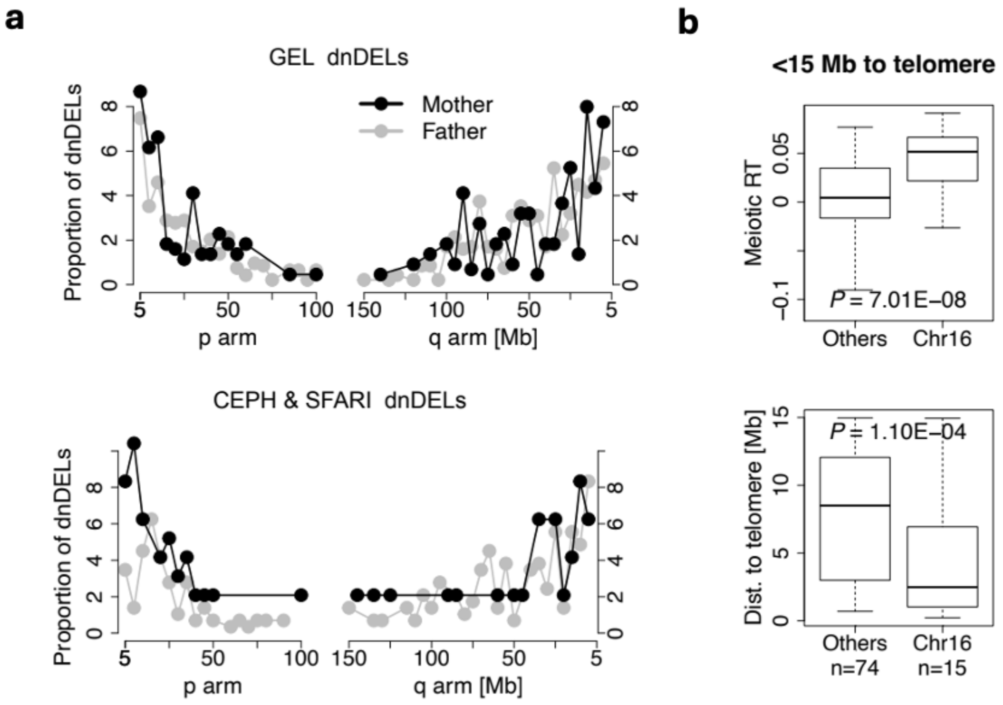

Figure S11. Enrichment of maternal dnDELs at subtelomeric, early replicating regions of chromosome 16. (a) Proportion of dnDELs as a function of the distance to telomere ends (5 Mb bins) in GEL (top) and in CEPH & SFARI cohort (bottom). (b) Comparison of replication timing (top) and distance to telomere ends (bottom) of dnDELs between chromosome 16 and the other chromosomes.

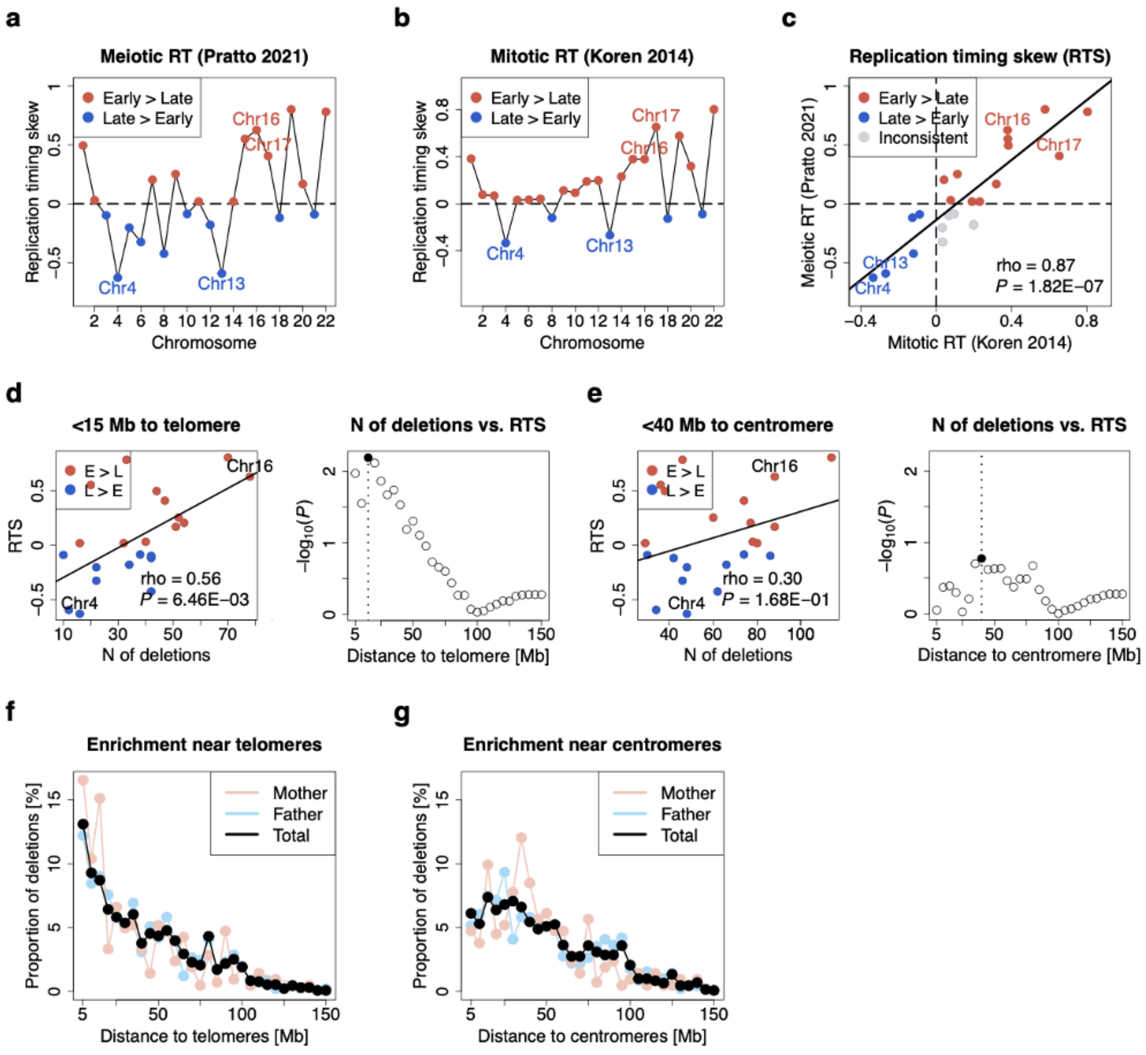

Figure S12. Analysis of dnSVs in regions of early and late replication timing and relative location to telomere and centromere. (a) Meiotic replication timing skew (RTS) derived from a male germline (Pratto et al., 2021). (**b**) Reference mitotic RT derived from lymphoblastoid cell lines. (**c**) Scatterplot showing replication timing skew (RTS) between germline and somatic cells. (**d)** Correlation between the number of deletions at the subtelomeric regions and RTS across chromosomes. (**e**) Correlation between the number of deletions at the centromeric regions and RTS across chromosomes. **(f)** Proportion of dnDELs as a function of the distance to telomere ends. **(g)** Proportion of dnDELs as a function of the distance to centromeres.

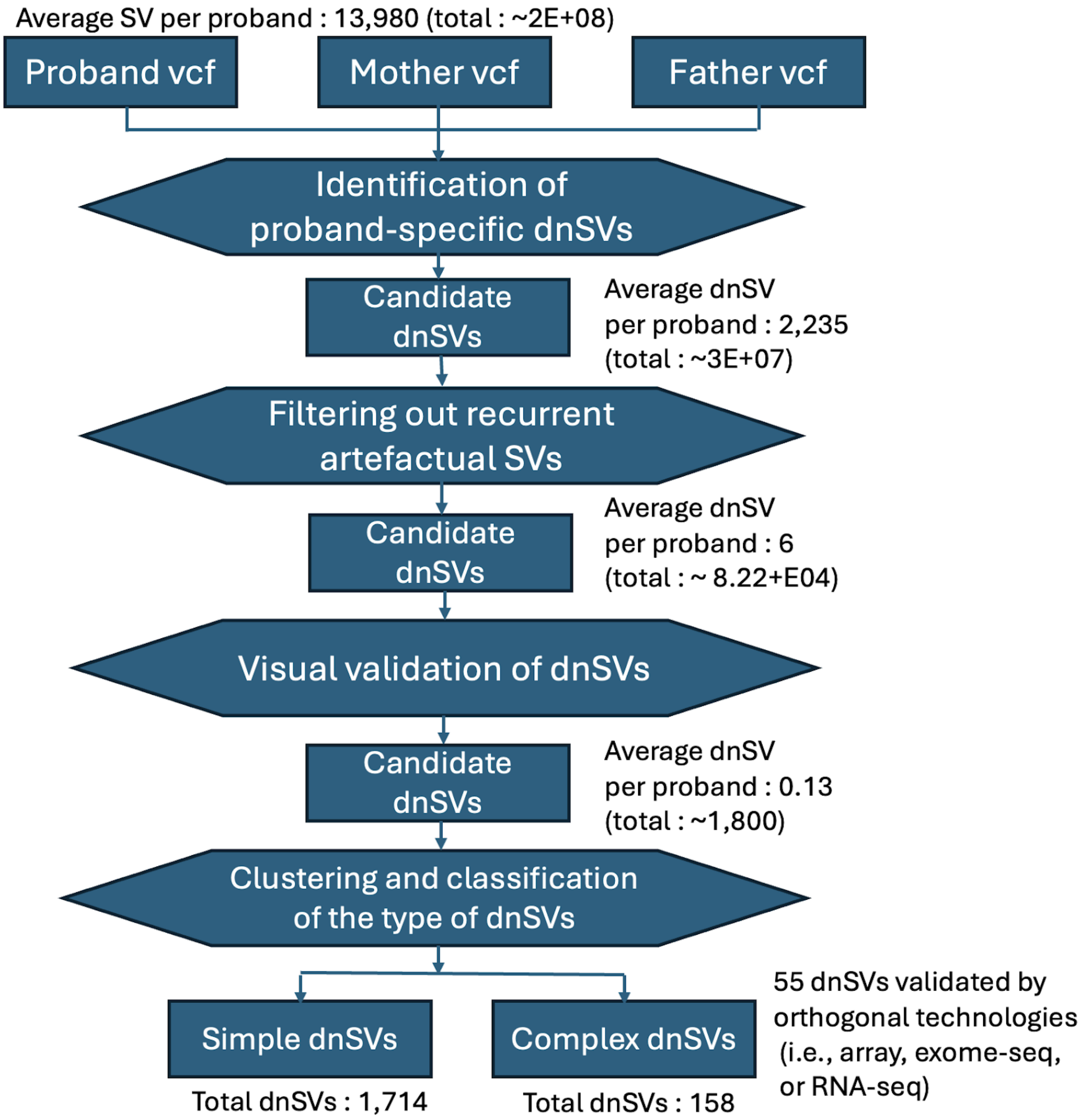

Figure S13. Flowchart for dnSV calling.
