# Supplementary material for: Complex de novo structural variants are an underestimated cause of rare disorders
Source: Nat Commun. 2025 Nov 3;16:9528. doi: 10.1038/s41467-025-64722-2 (PMC12583596; doi:10.1038/s41467-025-64722-2)
Supplement: Supplementary file 2 — Description of Additional Supplementary Files [file 41467_2025_64722_MOESM2_ESM.pdf]

Description of Additional Supplementary Files

Supplementary Data 1. Cohort description

Supplementary Data 2. Identified denovo SVs

Supplementary Data 3. List of duplications used in timing analysis

Supplementary Data 4. List of pathogenic CNVs identified by CANVAS
