# Supplementary material for: Complex de novo structural variants are an underestimated cause of rare disorders
Source: Nat Commun. 2025 Nov 3;16:9528. doi: 10.1038/s41467-025-64722-2 (PMC12583596; doi:10.1038/s41467-025-64722-2)
Supplement: Supplementary file 7 — Reporting Summary [file 41467_2025_64722_MOESM7_ESM.pdf]

nature portfolio

Corresponding author(s): Raheleh Rahbari

Last updated by author(s): 27/08/2025

Reporting Summary

Nature Portfolio wishes to improve the reproducibility of the work that we publish. This form provides structure for consistency and transparency in reporting. For further information on Nature Portfolio policies, see our Editorial Policies and the Editorial Policy Checklist.

Statistics

For all statistical analyses, confirm that the following items are present in the figure legend, table legend, main text, or Methods section.

n/a	Confirmed	
☐	☒	The exact sample size (*n*) for each experimental group/condition, given as a discrete number and unit of measurement
☐	☒	A statement on whether measurements were taken from distinct samples or whether the same sample was measured repeatedly
☐	☒	The statistical test(s) used AND whether they are one- or two-sided<br>*Only common tests should be described solely by name; describe more complex techniques in the Methods section.*
☐	☒	A description of all covariates tested
☐	☒	A description of any assumptions or corrections, such as tests of normality and adjustment for multiple comparisons
☐	☒	A full description of the statistical parameters including central tendency (e.g. means) or other basic estimates (e.g. regression coefficient) AND variation (e.g. standard deviation) or associated estimates of uncertainty (e.g. confidence intervals)
☐	☒	For null hypothesis testing, the test statistic (e.g. *F*, *t*, *r*) with confidence intervals, effect sizes, degrees of freedom and *P* value noted<br>*Give P values as exact values whenever suitable.*
☒	☐	For Bayesian analysis, information on the choice of priors and Markov chain Monte Carlo settings
☒	☐	For hierarchical and complex designs, identification of the appropriate level for tests and full reporting of outcomes
☐	☒	Estimates of effect sizes (e.g. Cohen's *d*, Pearson's *r*), indicating how they were calculated

Our web collection on statistics for biologists contains articles on many of the points above.

Software and code

Policy information about availability of computer code

Data collection: Data collection was carried out by Genomics England (https://www.genomicsengland.co.uk).

Data analysis: The cohort was whole-genome sequenced and read alignment and SV calling using Isaac and Manta, respectively, were performed by the Genomics England Bioinformatics team.

1.SVtools : https://github.com/hall-lab/svtools
2.Bedtools : https://bedtools.readthedocs.io/en/latest/
3.IGV : https://igv.org/
4.RepeatMasker : https://www.repeatmasker.org/
5.ClusterSV : https://github.com/cancerit/ClusterSV
6.samplot : https://github.com/ryanlayer/samplot
7.bamsnap : https://github.com/parklab/bamsnap
8.unfazed : https://github.com/jbelyeu/unfazed
9.Custom script : https://github.com/hj6-sanger/GEL_SV

For manuscripts utilizing custom algorithms or software that are central to the research but not yet described in published literature, software must be made available to editors and reviewers. We strongly encourage code deposition in a community repository (e.g. GitHub). See the Nature Portfolio guidelines for submitting code & software for further information.

Data

Policy information about availability of data

All manuscripts must include a data availability statement. This statement should provide the following information, where applicable:

- Accession codes, unique identifiers, or web links for publicly available datasets
- A description of any restrictions on data availability
- For clinical datasets or third party data, please ensure that the statement adheres to our policy

The data used in this study can be accessed via the Genomics England Research Environment, a secure cloud workspace. The raw data, including patient profiles and corresponding genomic sequencing data, are only available under restricted access for patient privacy reasons. Access can be obtained by first applying to become a member of either the Genomics England Research Network (https://www.genomicsengland.co.uk/research/academic) or the Discovery Forum (industry partners https://www.genomicsengland.co.uk/research/research- environment). The process for joining the network is described at https://www.genomicsengland.co.uk/ research/academic/join-gecip and consists of the following steps:
1. Your institution will need to sign a participation agreement available at https://files.genomicsengland.co.uk/documents/Genomics-England-GeCIP-Participation-Agreement-v2.0.pdf and email the signed version to gecip-help@genomicsengland.co.uk.
2. Once you have confirmed your institution is registered and have found a domain of interest, you can apply through the online form at https:// www.genomicsengland.co.uk/research/academic/join-gecip where you can specify the reason for access and expected timeframe that you wish to have access. Once your Research Portal account is created you will be able to login and track your application.
3. Your application will be reviewed within 10 working days.
4. Your institution will validate your affiliation.
5. You will need to complete the online Information Governance training and will be granted access to the Research Environment within 2 days of passing the online training.

Research involving human participants, their data, or biological material

Policy information about studies with human participants or human data. See also policy information about sex, gender (identity/presentation), and sexual orientation and race, ethnicity and racism.

Reporting on sex and gender	Sex metadata was provided by Genomics England (https://www.genomicsengland.co.uk). The gender information was used to analyze the effect of parents' age on de novo SV rate.
Reporting on race, ethnicity, or other socially relevant groupings	Not reported in this study.
Population characteristics	12,568 families, including 13,698 offspring with rare diseases, as part of the UK 100,000 Genomes Project, were analyzed in this study. All relevant metadata (e.g., age) were collected by Genomics England and can be accessed in an authorized manner.
Recruitment	The participants were recruited by Genomics England.
Ethics oversight	This research was made possible through access to the data and findings generated by the 100,000 Genomes Project. The 100,000 Genomes Project is managed by Genomics England Limited (a wholly owned company of the Department of Health and Social Care). The 100,000 Genomes Project is funded by the National Institute for Health Research and NHS England. The Wellcome Trust, Cancer Research UK and the Medical Research Council have also funded research infrastructure. The 100,000 Genomes Project uses data provided by patients and collected by the National Health Service as part of their care and support. This research was supported in part by the Wellcome Trust grant.

Note that full information on the approval of the study protocol must also be provided in the manuscript.

Field-specific reporting

Please select the one below that is the best fit for your research. If you are not sure, read the appropriate sections before making your selection.

[x] Life sciences [ ] Behavioural & social sciences [ ] Ecological, evolutionary & environmental sciences

For a reference copy of the document with all sections, see nature.com/documents/nr-reporting-summary-flat.pdf

Life sciences study design

All studies must disclose on these points even when the disclosure is negative.

Sample size	No Methods were used to determine sample size
Data exclusions	No data were excluded.
Replication	Genomic size and distributions of de novo deletions according to parent of origin

Randomization	N/A
Blinding	N/A

Reporting for specific materials, systems and methods

We require information from authors about some types of materials, experimental systems and methods used in many studies. Here, indicate whether each material, system or method listed is relevant to your study. If you are not sure if a list item applies to your research, read the appropriate section before selecting a response.

Materials & experimental systems

n/a	Involved in the study
☒	☐ Antibodies
☒	☐ Eukaryotic cell lines
☒	☐ Palaeontology and archaeology
☒	☐ Animals and other organisms
☐	☒ Clinical data
☒	☐ Dual use research of concern
☒	☐ Plants

Methods

n/a	Involved in the study
☒	☐ ChIP-seq
☒	☐ Flow cytometry
☒	☐ MRI-based neuroimaging

Clinical data

Policy information about clinical studies

All manuscripts should comply with the ICMJE guidelines for publication of clinical research and a completed CONSORT checklist must be included with all submissions.

Clinical trial registration	N/A
Study protocol	Data collection was carried out as detailed by Genomics England (https://www.genomicsengland.co.uk/initiatives/100000-genomes-project/documentation)
Data collection	Data collection was carried out as detailed by Genomics England (https://www.genomicsengland.co.uk/initiatives/100000-genomes-project/documentation)
Outcomes	N/A

Plants

Seed stocks	*Report on the source of all seed stocks or other plant material used. If applicable, state the seed stock centre and catalogue number. If plant specimens were collected from the field, describe the collection location, date and sampling procedures.*
Novel plant genotypes	*Describe the methods by which all novel plant genotypes were produced. This includes those generated by transgenic approaches, gene editing, chemical/radiation-based mutagenesis and hybridization. For transgenic lines, describe the transformation method, the number of independent lines analyzed and the generation upon which experiments were performed. For gene-edited lines, describe the editor used, the endogenous sequence targeted for editing, the targeting guide RNA sequence (if applicable) and how the editor was applied.*
Authentication	*Describe any authentication procedures for each seed stock used or novel genotype generated. Describe any experiments used to assess the effect of a mutation and, where applicable, how potential secondary effects (e.g. second site T-DNA insertions, mosiacism, off-target gene editing) were examined.*
